# Optimization and Performance Evaluation of Multi-Component Binder-Based Mortars Using Particle Packing Techniques

**DOI:** 10.3390/ma19051024

**Published:** 2026-03-06

**Authors:** Vanga Renuka, Sarella Venkateswara Rao, Tezeswi Tadepalli, Katarzyna Kalinowska-Wichrowska, Krzysztof Granatyr, Marta Kosior-Kazberuk, Małgorzata Franus, Adam Masłoń

**Affiliations:** 1 Department of Civil Engineering, National Institute of Technology Warangal, Warangal 506004, Telangana, India; vr721008@student.nitw.ac.in (V.R.); tezeswi@nitw.ac.in (T.T.); 2 Faculty of Civil Engineering and Environmental Sciences, Bialystok University of Technology, Wiejska 45E, 15-351 Bialystok, Poland; k.kalinowska@pb.edu.pl (K.K.-W.); krzysztof.granatyr@pb.edu.pl (K.G.); m.kosior@pb.edu.pl (M.K.-K.); 3 Department of General Construction, Lublin University of Technology, Nadbystrzycka 40 Street, 20-618 Lublin, Poland; m.franus@pollub.pl; 4 Department of Environmental Engineering and Chemistry, Rzeszow University of Technology, Powstańców Warszawy 12 Av., 35-029 Rzeszow, Poland

**Keywords:** multi-component binder, supplementary cementitious materials, D-optimal mixture design, packing density, drying shrinkage, eco-efficient mortar

## Abstract

**Highlights:**

**What are the main findings?**

- The D-optimal mixture design (DOD) method is used to determine the optimal material proportions.
- Proportioning of fine aggregate using the MTM method achieves max. packing density and min. void ratio.
- MCB-based mortars are able to attain their maximum strengths after 90 days.

**What are the implications of the main findings?**

- Maximum packing density is a reliable indicator for achieving mechanical and durability properties.
- Statistical mixture design and particle packing provide a systematic, optimized pathway.
- MCB systems substantially reduce energy consumption and CO_2_ emissions.

**Abstract:**

The use of a multi-component binder (MCB), consisting of Ordinary Portland Cement (OPC) combined with one or more supplementary cementitious materials (SCMs), has gained prominence for enhancing sustainability and improving the performance of cementitious systems. This study provides an integrated approach to optimize both binder composition and aggregate gradation through advanced mixture design and particle packing techniques. The MCB system consists of OPC partially replaced with SCMs such as fly ash (FA), Ground Granulated Blast Furnace Slag (GGBFS), metakaolin (MK), and silica fume (SF), with particle sizes ranging from micron to sub-micron scale. The D-optimal mixture design (DOD) method is used to determine the optimal material proportions by evaluating the relation between binder composition and wet packing density measured through the wet packing method (WPM). To further enhance packing efficiency, the Modified Toufar Model (MTM) is employed to optimize fine aggregate gradation. The maximum packing density is considered the primary criterion for identifying the optimal mix design, as it reflects the minimum void ratio and the most efficient particle size distribution. The optimized mortar mixes are evaluated for mechanical strength, pozzolanic reactivity, capillary water sorptivity, and drying shrinkage. Results indicate that the optimized MCB and optimized fine aggregate gradation improve the packing density and pozzolanic activity, significantly enhancing strength and durability performance. The incorporation of SCMs offers an effective strategy to improve performance while mitigating carbon emissions. Compared with C100, CFGMS-based systems achieved energy reductions of 35–40% and CO_2_ emission reductions of 34–48%.

## 1. Introduction

The cement manufacturing industry has to deal with the fact that it needs to meet global demand while also cutting down on CO_2_ emissions. In the last five years, direct CO_2_ emissions from making cement have remained the same, with a minor increase of 1% in 2022. On the other hand, the sector needs to cut its CO_2_ intensity by 4% every year until 2030 in order to meet the Net Zero Emissions by 2050 [1,2]. To reach this goal, it is important to lower the clinker-to-cement ratio by using clinker alternatives, making energy use more efficient, using low-carbon fuels, making materials more efficient, and using new technologies like Carbon Capture and Storage (CCS). Using Supplementary Cementitious Materials (SCMs) instead of Ordinary Portland Cement (OPC) is a good way to lower CO_2_ emissions and their adverse impact on the environment, and it is a common and effective solution [3,4,5]. Some of the SCMs used in concrete are fly ash (FA), Ground Granulated Blast Furnace Slag (GGBFS), limestone, silica fume (SF), and metakaolin (MK). These materials decrease the amount of clinker in cement, which lowers the carbon footprint due to the energy-intensive nature of clinker manufacture [6,7]. SCMs exhibit a more gradual pozzolanic reaction compared with cement, leading to a delayed strength development in the initial phase [8,9]. Researchers have successfully utilized these industrial by-products in concrete, either by incorporating them into cements or by batching them separately into concrete, thereby reducing the carbon footprint and fostering sustainability [10]. However, when compared with OPC, the long-term durability benefits of SCMs overcome this drawback. Consequently, the use of multi-component binders, which consist of OPC combined with one or more SCMs, is being standardized in accordance with the latest advancements in cement production [11]. SCMs vary in mineral composition and physical properties. Improved mechanical characteristics of cement slurry improve the performance of mortar and concrete. Results from the interaction processes between these components, which in turn lead to composite SCMs with specific Ca-Si-Al ratios. As a result, SCMs are increasingly being utilized to partially replace OPC, not only to improve sustainability but also to enhance the packing density of the binder system. Research concerning multi-blended cementitious systems, including several SCMs with particle sizes varying from micron to nano levels, has been rarely addressed.

Concrete, a composite material consisting of cementitious materials (such as OPC, FA, GGBFS, MK, SF, etc.) and aggregates (such as sand, gravel, crushed stone, etc.), has a granular skeleton and water that hardens with time. Packing density, which is the ratio of solid volume to bulk volume, is a fundamental property that describes many granular systems [12]. Inevitably, the characteristics of the resulting composite material are determined by the particle packing behavior of the granular components. For more than a century, researchers from a variety of fields have shown an interest in particle packing [13]. Previous studies show that systematic proportioning of aggregates using particle packing techniques improves concrete performance [14]. To obtain the maximum packing density while reducing voids, aggregates are proportioned in a manner that fills spaces between larger aggregates with smaller aggregates. Optimizing the proportions of various component materials may enhance the packing density of concrete mixes. A decrease in voids enhances aggregate packing density, thereby reducing the necessary OPC content, thus lowering the environmental impact of concrete [15]. Using particle packing models (PPMs) has been shown to significantly lower the cement content of concrete by strengthening the skeleton of aggregates [16]. Increasing the particle packing density in cementitious materials can indeed help reduce the quantity of paste required to fill voids, as the particles occupy space more efficiently. Additionally, improved packing can enhance the rheology of the mixture, requiring less paste for lubrication, which may reduce the overall water-to-binder ratio and improve the strength and durability of the final product [17,18]. Measuring packing density of binder particles, especially fine particles (less than 100 μm), is more challenging than measuring aggregate particle packing due to uncertainty in the measurement of inter-particle forces and compaction degree. Cementitious materials often display significant inter-particle interactions, including hydration effects, electrostatic forces, and Van der Waals forces. When particles cluster or agglomerate, these forces can make it difficult to determine the actual packing density because of uneven packing. Surface interactions and compaction effects are more likely to occur with fine particles than with coarse aggregates due to their larger surface area-to-volume ratio. As moisture content, handling methods, and confinement affect the packing density measurements, fine particle compaction is difficult to achieve. To address this difficulty, the wet packing method (WPM) is implemented to determine the packing density of cementitious materials. WPM identifies the highest solid concentration, as it is an inherent characteristic of any binder system regardless of water content [19,20,21,22]. The fineness of SCMs impacts their ability to fill voids or improve packing. With a broader particle size distribution (PSD), medium particles would fill the voids between large particles, fine particles would fill the voids between medium particles, and very fine particles would fill the voids between fine particles, and so on, removing more voids, which indeed increases the packing density [21,23].

Optimization of cement and concrete mixtures is a focus of research to balance strength, durability, and workability, while also addressing sustainability concerns. Design of Experiments (DOE) is a well-known approach that employs statistical analysis, ensuring that valid results are efficiently obtained with minimal effort, time, and resources. DOE helps researchers to explore the effects of many factors in an efficient way by carefully planning and analyzing experiments. This facilitates the identification of significant variables and their interactions, resulting in optimum solutions without requiring extensive individual tests. The key experimental design techniques used in optimizing construction material design are Response Surface Methodology (RSM) [24,25,26,27,28,29], the Taguchi Method [30,31,32,33], Factorial Design [34], the Mixture Design Approach [35,36], and Machine Learning Models [34]. The primary goal of these approaches is to forecast the response variables with fewer tests. D-optimal mixture design (DOD) is a recognized and efficient technique that simultaneously optimizes many objectives, reduces the number of tests required, and provides highly accurate predictions. It is particularly effective when variables exhibit interdependencies among variables and when constraints are imposed [28,35,37]. Recent studies have highlighted the growing importance of D-optimal mixture design in optimizing the synergy among multiple SCMs, ensuring better mechanical performance, durability, and environmental efficiency [38,39,40]. Some researchers also emphasize that fine-tuning proportions with this design approach helps improve setting time and reduce water demand, which can be challenging with materials like MK and SF due to their high surface area and reactivity challenges.

**Figure 1:**
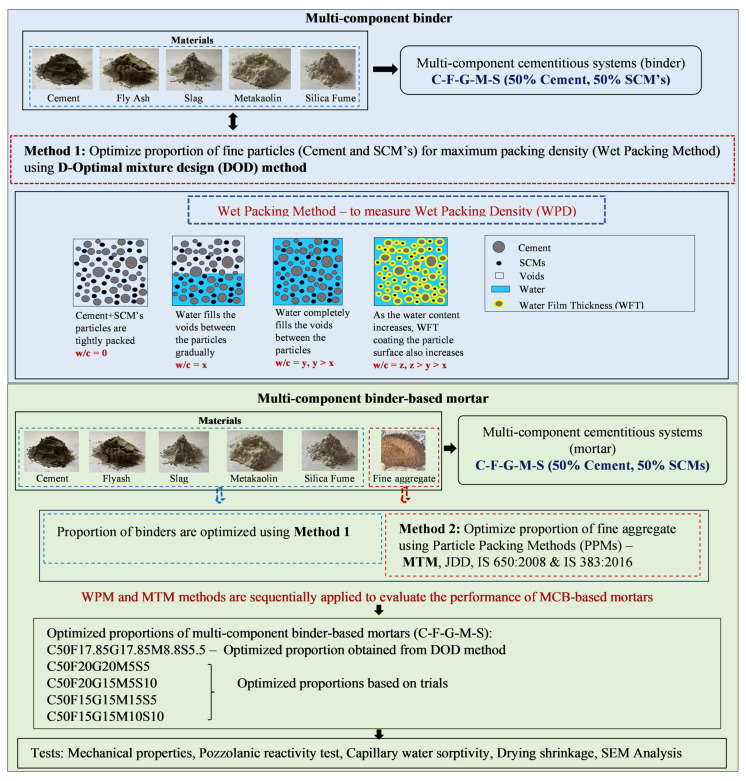
The framework for the study.

The current experimental investigation is primarily concerned with substituting 50% of OPC with the potential use of SCMs such as FA, GGBFS, MK, and SF to form multi-component cementitious systems. Because of the significant impact that water plays in determining the packing density of SCMs (owing to the surface forces present), the mix design procedure in this study suggests that fine materials (SCMs) and fine aggregate packing densities be considered independently. The need of the hour is to understand the effects of multi-sized pozzolanic materials combined with OPC in the form of multi-component cementitious systems through the particle packing approach. Therefore, based on the above, an effective strategy in this study for optimizing multi-component binders (MCB) is the integration of packing density theory with the D-optimal design (DOD) method. In order to develop high-performance mixes, this complementary approach combines the best features of statistical design with those of material science. The following are its advantages: (1) Considering the effects of both solid and liquid stages at the same time is more accurate than models that solely enhance the packing of solid granular particles; (2) the intrinsic characteristics of MCB can be effectively employed through the optimization of close packing theory in its development. In particular, the DOD method is employed to initially establish the functional relation among materials and packing density. The optimum packing density is then considered as a measure in determining the optimal content of OPC, FA, GGBFS, MK, and SF. Further, mortar studies are performed by proportioning fine aggregates through the Modified Toufar Model (MTM), J. D. Dewar (JDD), and IS 650:2008 [41] methodologies, and the resulting proportions are then compared with fine aggregates as specified by IS 383:2016 [42]. The optimized mortar mixes are evaluated for time-dependent properties such as mechanical strength, pozzolanic reactivity, capillary water sorptivity, and drying shrinkage. Finally, the environmental impact assessment for the developed mixes was performed. The framework for the present study is shown in Figure 1.

This interdisciplinary research focuses on the analysis of complex relationships and interactions between multicomponent pozzolanic materials, water, and the solid phase in cementitious systems, which have not yet been systematically and quantitatively identified or clearly described in the literature. The combined application of the WPM, DOD, and MTM represents a methodological advancement by coupling packing theory with statistical mixture optimization and experimental validation. Unlike traditional approaches that treat packing density and compositional design separately, this integrated framework enables simultaneous control of PSD, volumetric solid concentration, and multi-component interactions within a unified design strategy. The proposed research approach fills a significant research gap, going beyond the traditional framework of civil engineering and also contributing to the field of environmental engineering through the rational use of industrial by-products and waste, such as FA, GGBFS, MK, and SF, as valuable binder components. This approach enables a reduction in the use of Portland cement clinker and, consequently, a reduction in the energy consumption of technological processes and CO_2_ emissions, which is consistent with the principles of a circular economy. Consequently, the proposed solutions integrate material, technological, and environmental aspects, providing a novel approach for the design of high-performance and low-emission cementitious binders that address the current challenges of sustainable development in construction.

## 2. Materials Characterization and Methods

### 2.1. Materials

**Figure 2:**
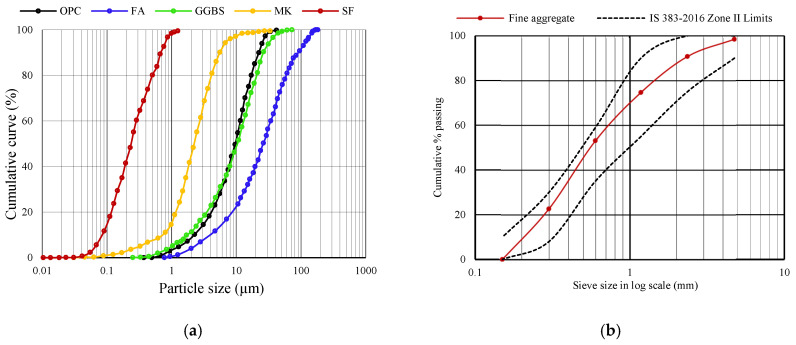
PSD of raw materials: (**a**) OPC and SCMs and (**b**) fine aggregate.

**Figure 3:**
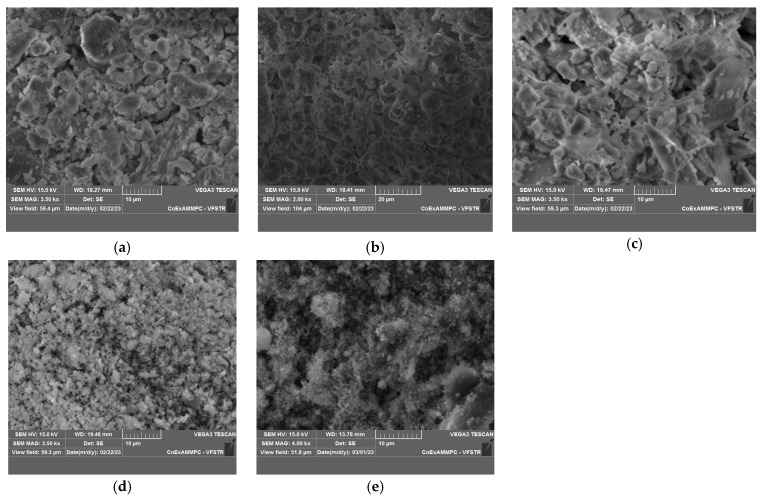
SEM images of (**a**) OPC, (**b**) fly ash, (**c**) GGBFS, (**d**) MK, and (**e**) SF.

**Figure 4:**
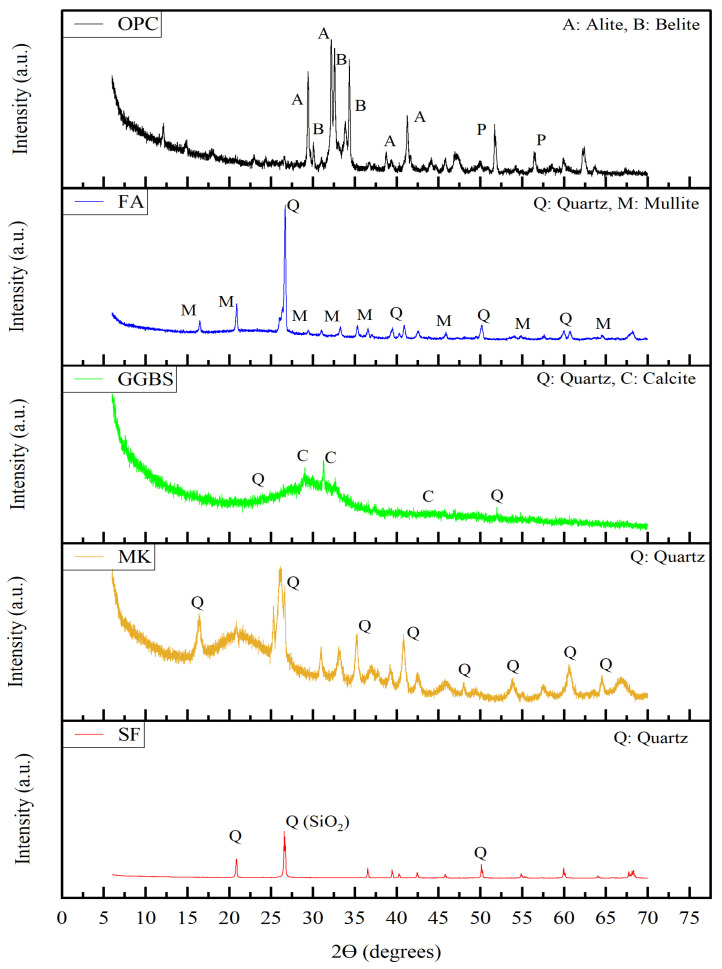
XRD pattern of OPC, FA, GGBFS, MK, and SF.

OPC 53 Grade, satisfying the guidelines of IS 12269:2013 [43], equivalent to ASTM C150/C150M [44], is utilized to prepare a multi-component binder and mortar by interblending it with SCMs. FA, GGBFS, MK, and SF are used as SCMs as partial replacements for OPC. The chemical compositions of the raw materials determined by X-ray Fluorescence (XRF) are listed in Table 1. The PSDs of these materials are measured using a Malvern^®^ laser particle size analyzer (Malvern Panalytical, Malvern, UK), as shown in Figure 2. Surface morphology of the materials is analyzed by Scanning Electron Microscopy (SEM) (ZEISS, Oberkochen, Germany), as shown in Figure 3. The X-ray diffractometer (XRD) patterns of OPC, FA, GGBFS, MK, and SF obtained are depicted in Figure 4.

The high CaO content in OPC is consistent with the presence of calcium silicate phases (alite and belite) observed in XRD. The moderate SiO_2_ content combines with CaO to form these clinker minerals. The high SiO_2_ and Al_2_O_3_ contents in FA explain the presence of quartz and mullite. The low CaO confirms that FA is a low-calcium pozzolan. The amorphous hump corresponds to the reactive aluminosilicate phase, which is responsible for its pozzolanic activity. The high aluminosilicate content in MK explains the strong pozzolanic potential. The absence of crystalline kaolinite peaks confirms successful calcination to amorphous. The very high SiO_2_ content in SF corresponds to predominantly amorphous silica. The broad hump confirms its non-crystalline structure, which explains its very high pozzolanic reactivity. The predominance of amorphous phases in FA, GGBS, MK, and SF suggests high reactivity in blended systems, which is expected to enhance secondary hydration reactions and microstructural densification.

Natural river sand of Zone II conforming to IS 383:2016 [42], equivalent to ASTM C33/C33M-18 2018 [45], was used in this study. The fineness modulus, specific gravity, and bulk density of fine aggregate were determined to be 2.6, 2.61, and 1582 kg/m^3^, and the grain size distribution is shown in Figure 2b. Surface parameters such as roughness, sphericity, and angularity play a crucial role in proportioning aggregates utilizing particle packing techniques as they impact the packing degree. Portable water with pH = 7.12 was used for casting and curing specimens.

**Table 1:** Chemical composition and physical properties of OPC and SCMs (adopted from [46]).

	Chemical Composition (wt. %)										Physical Properties		
	CaO	Al_2_O_3_	SiO_2_	Fe_2_O_3_	MgO	SO_3_	Na_2_O	K_2_O	Cl Ion	Loss	Specific Surface Area (m^2^/kg)	Density (g/cm^3^)	Particle Shape
OPC	64.1	5.8	20.2	3.23	-	2.66	0.15	1.2	0.006	1.416	340	3.15	Angular, irregular
FA	1.79	26.37	56.15	6.44	2.35	0.056	1.1	3.8	0.09	1.832	370	2.2	Spherical, regular
GGBS	37.92	13.27	37.97	1.16	5.64	0.23	0.84	0.56	0.015	0.56	420	2.78	Angular, irregular
MK	0.45	38.11	51.59	1.82	0.23	0.14	0.11	0.43	-	1.33	1200	2.6	Plate-like, lamellar
SF	0.71	0.58	91	0.24	0.33	-	0.29	1.26	-	1.58	20,200	2.25	spherical

### 2.2. Mathematical Modelling of the Mix Design

A D-optimal mixture design (DOD) is a custom design that is used in cases where classical designs (like full factorial or simplex designs) are impractical or inefficient. DOD is a statistical approach in experimental design with constrained conditions that specifically focuses on optimizing mixtures while minimizing the number of experimental trials [28]. DOD operates under the condition that the total of variables must be 100% (or 1 if normalized), and the ratio of variables must be between 0 and 1. This ensures that all components are utilized within their permissible limits and establishes the bounds for each variable.

**Figure 5:**
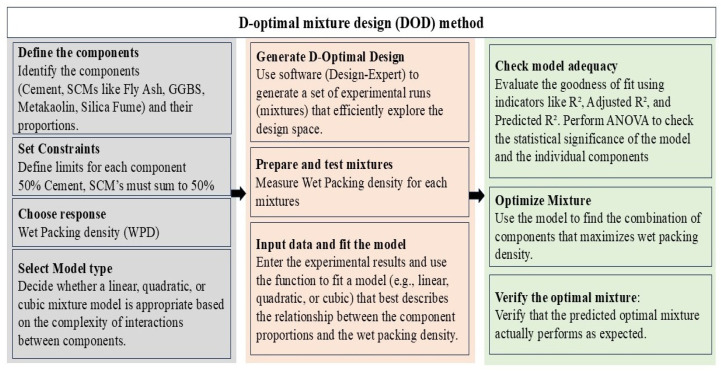
Typical D-optimal mixture design (DOD) method.

In this study, DOD was employed to develop the functional relation between materials and packing density. The mixture components OPC, FA, GGBFS, MK, and SF were considered as independent variables (the sum total of their contents is 100%). The response variable is packing density, which is used to evaluate and optimize the binder composition. Table 2 displays the variable constraint requirements and response. These constraints are used to restrict the mixture design space and test only practical and realistic mix proportions. The Scheffe Quadratic model is a common choice in mixture designs, and it is typically used to relate the proportions of the components to a response variable (packing density), as shown in Equation (1).

(1) $$E\left(y\right)=\sum_{i=1}^{q}\beta_{i}x_{i}+\sum_{i=1}^{q-1}\sum_{j+1}^{q}\beta_{ij}x_{i}x_{j}$$

where E(y) is the expected response value, $\beta_{i}$, and $\beta_{ij}$ denote the constant coefficients, and $x_{i}$ and $x_{j}$ are the variables. Twenty-four experimental runs were designed based on DOD method within the statistical design program Design-Expert 13, and the typical DOD approach is shown in Figure 5. Upon completing the design, the experimental runs in the design matrix are examined. Each run will have a fixed X_1_(A) = 0.5, while the proportions of X_2_(B), X_3_(C), X_4_(D), and X_5_(E) will fluctuate based on DOD, ensuring that the total of these four components remains 0.5. Data from the experiments are reported in Table 3. Through an analysis of variance (ANOVA), the competence of the model that has been developed was verified. Finally, this approach helps to optimize the ratios of SCMs to form an MCB with maximum packing density.

## 3. Experimental Program

### 3.1. Preparation of Multi-Component Binder Systems

This study focuses primarily on optimizing the packing density of MCB to find the most efficient arrangement of particles to achieve maximum packing density. Twenty-four experimental runs (see Table 3) were designed based on the DOD method, and the volumetric replacement ratios were considered while performing the test. The test determines the solid concentration of combinations at varied water-to-binder ratios (w/b)_vol_ using a cylindrical mold filled with paste and consolidated on a vibrating table for 60 s. Initial studies were carried out to determine the packing density of cementitious materials, beginning with a relatively low w/b ratio and gradually increasing the w/b ratio until the solid concentration reaches a maximum value and subsequently decreases. Based on the initial trials conducted, it was chosen to maintain a constant (w/b)_vol_ ratio of 0.6 and an SP dosage of 1% for all combinations of binder to provide a baseline for comparison. Polycarboxylate Ether (PCE)-based superplasticizer (SP) is highly effective in breaking agglomerates by providing electrostatic repulsion and steric hindrance, ensuring smaller particles are better dispersed to fill the voids between the larger ones.

### 3.2. Optimization of Fine Aggregate by Using Particle Packing Methods in Multi-Component Binder-Based Mortars

Once an optimized combination of MCB systems is determined using the DOD method, the study focuses on characterizing the mechanical properties, pozzolanic reactivity (Strength Activity Index), and capillary water sorptivity of multi-component binder-based mortar (using the concept of particle packing). In addition, its effect on the drying shrinkage properties up to 180 days is investigated in this study.

#### 3.2.1. Proportioning of Fine Aggregates

The proportions of various sizes of sand that pass through a 4.75 mm aperture are determined using the MTM [47,48], JDD [48], IS 650:2008 [41], and IS 383:2016 [42] methods, and subsequently, these proportions are incorporated into mortar blends to investigate the packing density using WPM [49].

#### 3.2.2. Preparation of Multi-Component Binder-Based Mortar Systems

An optimized combination of multi-component binders obtained from the DOD method is combined with fine aggregate in the predetermined proportions, and also the combinations obtained from the trial method are considered in the study (see Table 4). The mortar mixes are maintained with a w/b ratio of 0.4 and a binder-to-sand ratio of 1:3 in order to assess the influence of various SCMs on the mechanical properties of the mortar. SP of 1% (by wt. of binder) is used to improve the properties of the mortar. Various steps are performed in a planetary mortar mixer to prepare the mortar. To achieve a homogenous consistency, a mixture of OPC and SCMs is initially mixed dry, and then water and SP are added to the mixing container. Subsequently, the fine aggregate is gradually incorporated into the cement paste and mixed at 140 rpm for 2 min. The mixing speed is increased to 285 rpm for further homogenization of the mortar. This approach lowers the possibility of producing clumps or agglomerations of cement and SCMs, and it allows for a more homogeneous paste before the fine aggregate is added. Mortar cubes with an edge of 70.6 mm were cast and demolded after 24h, then cured in water (20 ± 1 °C) and tested at the ages of 3, 7, 28, and 90 days [49].

## 4. Test Methods for Multi-Component Cementitious Systems

### 4.1. Wet Packing Method (WPM)

Homogenized materials and water were added to the paste mixer in accordance with the mixing procedure outlined in Section 3. The mass and volume of the blended cementitious paste in the mold are denoted as M and V, respectively. From the test results obtained, the Wet Packing Density (WPD) of the cementitious materials was determined using Equation (2) as follows:

(2) $$\varphi =\frac{V_{c}}{V}$$

where $V_{c}$ refers to the total solid volume of the cementitious materials in the mold. When the cementitious materials include several components designated as x, y, and z, respectively, the solid concentration is determined as shown in Equation (3):

(3) $$V_{c}=\frac{M}{{\rho_{w}µ}_{w}+{\rho_{x}R}_{x}+{\rho_{y}R}_{y}+{\rho_{z}R}_{z}}$$

where $\rho_{x}$, $\rho_{y}$, and $\rho_{z}$ are the solid particle densities of materials, $\rho_{w}$ is the density of water. $R_{x}$, $R_{y}$, and $R_{z}$ are the volumetric ratios of a, b, and c to the total binder content, where $R_{x}$ + $R_{y}$ + $R_{z}$ = 1. The highest value of φ achieved is identified as the packing density ($\varphi_{max}$) of the cementitious systems.

The minimum voids ratio ($e_{min}$), which is the ratio of the minimum voids volume to the solid volume of the particles obtained from the measured $\varphi_{max}$, can be determined using Equation (4):

(4) $$e_{min}=\frac{1-\varphi_{max}}{\varphi_{max}}$$

where $e_{min}$ and $\varphi_{max}$ are void ratio and packing density, respectively.

### 4.2. Particle Packing Methods for Proportioning of Fine Aggregate

The determination of the mechanical and workability properties of mortar relies significantly on the PSD of fine aggregate, as it comprises a major percentage of material in mortar. The Modified Toufar Model (MTM) and J D Dewar (JDD) models are employed to get an accurate classification of fine aggregate. The MTM method is a technique that determines the packing density of a mixture of aggregates based on the packing density and mean diameter of individual-size aggregate fractions. When blending two distinct aggregate sizes, the smaller size should be used first because it has a higher ratio of fine to coarse aggregates, whereas the JDD model is a technique that determines the void ratio of a polydispersed aggregate system by using the mean diameter and void ratio of aggregate fractions individually. The details of particle packing methods have been discussed by several researchers [14,49,50].

### 4.3. Mechanical Properties

In order to investigate the mechanical properties of multicomponent cement-based mortar samples, a compression test was conducted according to IS 4031 (Part 6) [51]. Mortar cubes with an edge of 70.6 mm were cast and demoulded after 24 h, then cured in water (20 ± 1 °C) and tested at the ages of 3, 7, 28, and 90 days. Flexural strength test was performed on 40 mm × 40 mm × 160 mm mortar samples after curing periods of 3, 7, 28, and 90 days. A flexural strength was assessed using a three-point load test at a loading rate of 0.1 mm/min as per ASTM C348 [52].

### 4.4. Pozzolanic Reactivity Test—Strength Activity Index (SAI)

The pozzolanic reactivity was evaluated using the Strength Activity Index (SAI) in compliance with ASTMC311/C311M-22 [53] and as indicated by ASTM C618 [54]. The SAI value was determined by evaluating the compressive strength of cube mortar samples measuring 70.6 mm × 70.6 mm × 70.6 mm in accordance with IS 4031-Part 6 [51]. Strength activity index (SAI) of the blended cement mortar is expressed as the ratio of compressive strength of blended cement mortar cubes to that of the control mix, as shown in Equation (5):

(5) $$SAI\left(\%\right)=\frac{\mathrm{Compressive}\;\mathrm{strength}\;\mathrm{of}\;\mathrm{blended}\;\mathrm{mortar}}{\mathrm{Compressive}\;\mathrm{strength}\;\mathrm{of}\;\mathrm{control}\;\mathrm{mortar}}\times 100$$

### 4.5. Capillary Water Absorption

The absorption rate in cement-based systems, also known as sorptivity, which illustrates the inter-connectivity of pores inside the matrix, is a common way to assess the resistance of concrete or cement mortar to moisture penetration through capillary absorption. This test is conducted in accordance with ASTM C 1585 [55] codal requirements. Two-disc specimens (50 mm in diameter and 25 mm in height) were prepared for each mix, and the test was performed after 56 days of curing. In this test method, specimens were oven-dried at 105 °C for 24 h and cooled at room temperature for 1 day. By immersing the bottom surface of cement mortar specimens in water, the capillary water absorption rate was determined, while the remaining surfaces were coated with epoxy resin. Water levels in the test apparatus were maintained at a distance of approximately 2–3 mm from the bottom of the specimens as outlined in the standard method. The capillary absorption coefficient $A_{c}$ can be calculated using Equations (6) and (7):

(6) $$\Delta W=\frac{m_{0}-m_{1}}{S}$$

where ΔW is the absorbed water amount (g/m^2^). $m_{0}$ and $m_{1}$ are the weights of the disc samples without and with water penetration (g), and S is the water absorption area (m^2^).

(7) $$\Delta W{=A}_{c}\sqrt{t}$$

where t is the water absorption time (hours). Furthermore, an increased value of $A_{c}$ corresponds to an increased rate of water absorption in the cement composites.

### 4.6. Drying Shrinkage

Drying shrinkage of mortar specimens (25 mm × 25 mm × 285 mm) is determined by measuring the change in length with a length comparator ASTM C490/C490M [56] following the guidelines of ASTM C157/C157M-17 [57]. Immediately following 28 days of curing at a surface-dry state, the initial comparator reading is recorded. Subsequently, specimens are subjected to the ambient drying condition at a temperature of 27 ± 3 °C and a relative humidity of 50 ± 5%. The length variation is assessed at 3, 7, 14, 28, 56, 90, 120, and 180 days. Shrinkage strains $\left(S_{d}\right)$ of specimens are determined using Equation (8):

(8) $$S_{d}=\;\frac{L_{i}-L_{x}}{G}\times 100$$

where $L_{i}$ and $L_{x}$ represent the initial comparator reading and the comparator reading at a certain exposure duration, respectively. G represents the gauge length, 250 mm.

## 5. Results and Discussions

### 5.1. Model Adequacy Analysis

In this study, 24 experimental runs were developed with the D-optimal mixture design method to assess the effect of the interactions between fine particles (OPC, FA, GGBS, MK and SF) on the wet packing density of MCB. Considering the testing data, a Scheffe regression model was developed and the corresponding regression equation for packing density represented by Equation (9):

(9) $$y=0.335\left(B\right)+0.887\left(C\right)-1.685\left(D\right)-2.816\left(E\right)+5.391\left(B\right)\left(C\right)+15.636\left(B\right)\left(D\right)+14.580\left(B\right)\left(E\right)\;+8.122\left(C\right)\left(D\right)+12.572\left(C\right)\left(E\right)+16.164\left(D\right)\left(E\right)$$

where y is packing density, B (FA), C (GGBS), D (MK), and E (SF) are varying factors, and A (OPC) is a fixed factor = 0.5. When a variable (like OPC) is held constant in Design Expert version 13, the software does not explicitly estimate its coefficient. This is because the constant variable is already incorporated into the model implicitly, and its effect is accounted for as part of the baseline or interaction terms. Furthermore, the response is predicted using Equation (9) for specific levels of each factor. Fixing one variable does not intrinsically change the optimality of the design as long as the responses are measured precisely and other variables are varied within their bounds.

Following the data gathered for the experiments, terms with a *p*-value greater than 0.05 were deemed insignificant and removed from the model, and the final model was developed from the remaining significant terms. The statistical significance was evaluated using analysis of variance (ANOVA), with findings shown in Table 5. Notably, the Model F-Value of 37.00 (Fisher’s variance ratio) denotes a statistically significant model. Among the terms, BC, BD, BE, CD, CE, and DE were identified as significant contributors to the model. The F-value exceeds the *p*-value, which is below 0.05, indicating that the independent variables exhibit notable variations in packing density at a 95% confidence level. As a result, the model shows statistical significance. The lack of fit F-value of 3.28 indicates that it is not significant compared with pure error, and the Pre-R^2^ (0.829) value aligns reasonably with Adj-R^2^ (0.933).

**Figure 6:**
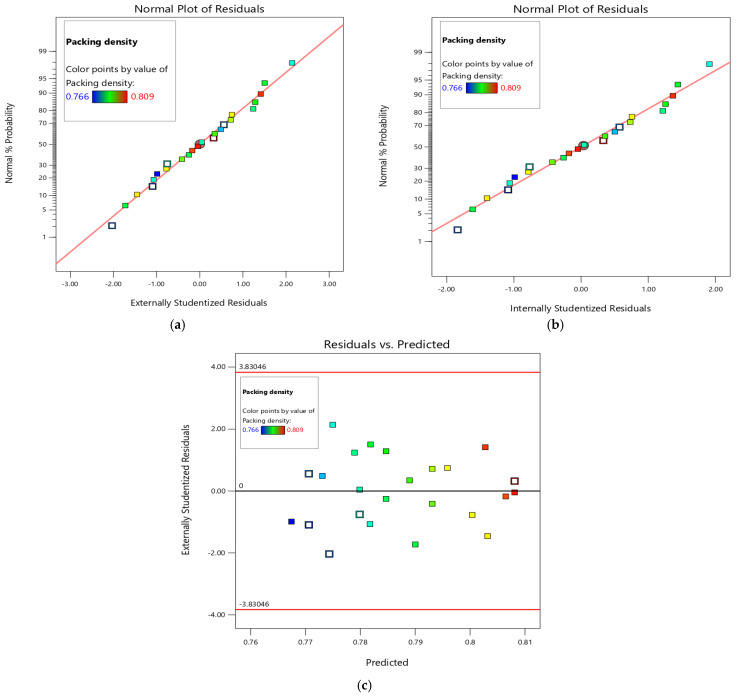

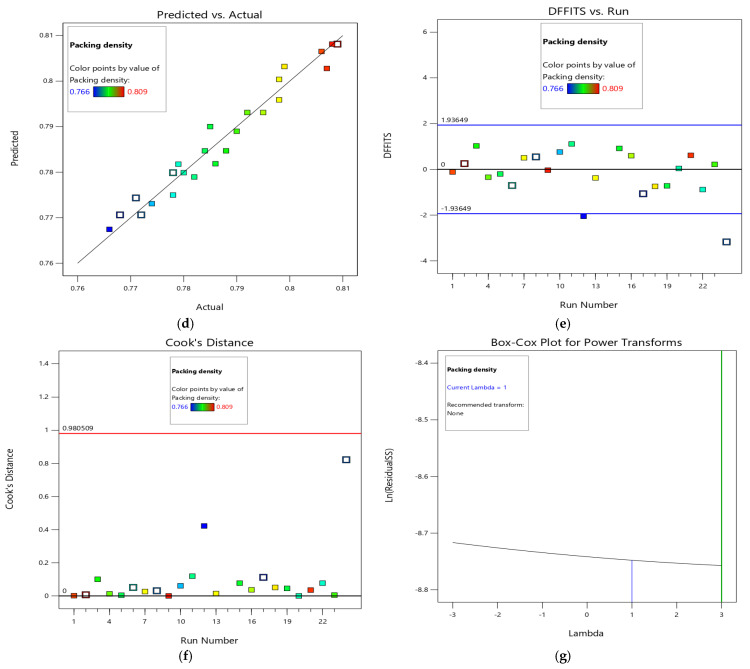
Diagnostics of the built Scheffe model: (**a**) normal plot of residuals (external), (**b**) normal plot of residuals (internal), (**c**) residuals vs. predicted, (**d**) predicted vs. actual, (**e**) Plot of DFFITS versus run number, (**f**) Cook’s distance vs. run number, (**g**) Box—Cox plot for power transform.

A residual normality plot, a residual vs. predicted plot, and a predicted vs. actual plot (see Figure 6) are some of the diagnostic graphs that were used to determine if the proposed quadratic models are appropriate and adequate to test the reliability of the model in this study. Figure 6a,b show that the residuals of packing density are aligned along a diagonal line, signifying adherence to a normal distribution, hence confirming the randomness of the residuals and suggesting that the employed quadratic model is realistic. The residual data points closely align with the diagonal line, exhibiting little variation in the normal % probability plots, indicating a minimal normal error distribution of the response variables [58]. If the random normal distribution of residuals is between +3 and −3, and if +2 and −2 sigma values are within the set limits, the used quadratic model is sufficient, reliable, and valid [59]. Figure 6c reports that the residuals of packing density were randomly distributed around a residual value of 0. Figure 6d compares the actual experimental results with the expected values derived from the proposed multiple regression model. The significant correlation between predicted and experimental response values is shown by the data points arranged close to the diagonal line [60]. Figure 6e shows that the DFFITS values corresponding to all response points are confined within the range of +2 and −2, indicating that the predicted data may be impacted by experimental data. Cook’s distance helps forecast the influence of certain data points to identify outliers. For the quadratic model to be considered valid and precise, all points must reside inside the interval 0 to 1. As evident in Figure 6f, the variations in packing density for all data points remain within the defined Cook’s points, indicating that none of the data points adversely affects the quadratic models. The packing density’s lambda value is 1, as shown in Figure 6g, signifying that no modification is necessary, since the quadratic model is precise and exhibits a robust fit for the chosen quadratic models [61].

#### Effect of Variables on Packing Density

**Figure 7:**
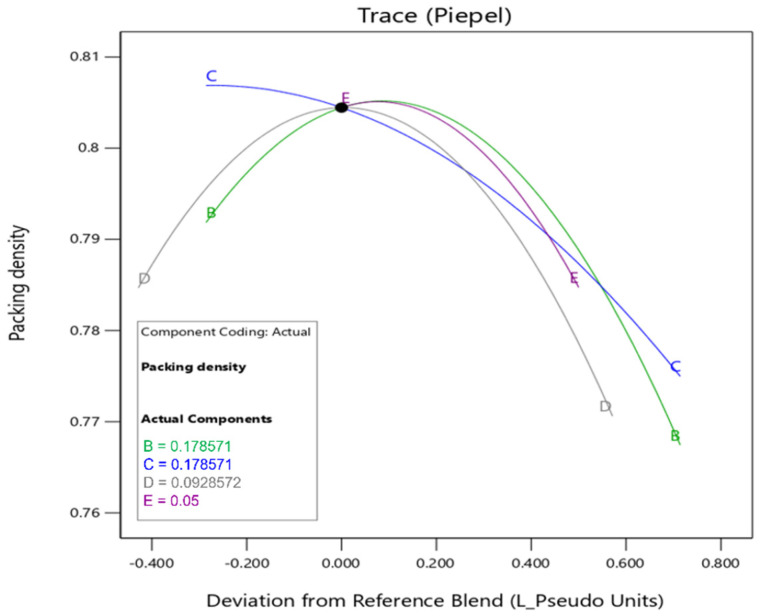
Trace plot—Effect of variation on MCB packing density.

The response trace plot in Figure 7 shows how parameters affect the packing density of MCB. The trace plot typically shows multiple lines, where each line represents the effect of a particular factor (or variable) on the response. This plot helps to visualize how the response variable changes when you adjust one factor at a time while keeping other factors constant. The curves illustrate the sensitivity of the developed model to any variations of the variables (OPC, FA, GGBFS, MK, and SF). From Figure 7, it is observed that the SCMs with multi-sized particles show a pronounced fluctuation range, signifying a notable impact on the packing density of the binder that fits with the ANOVA results. It is clear that optimal percentages of different SCMs can make the packing density of the multi-component binder better. SF (because of fine particles) tends to agglomerate in the cementitious system when the quantity exceeds a specific threshold. Moreover, excessive addition of SF decreases the packing density with an increase in water demand.

**Figure 8:**
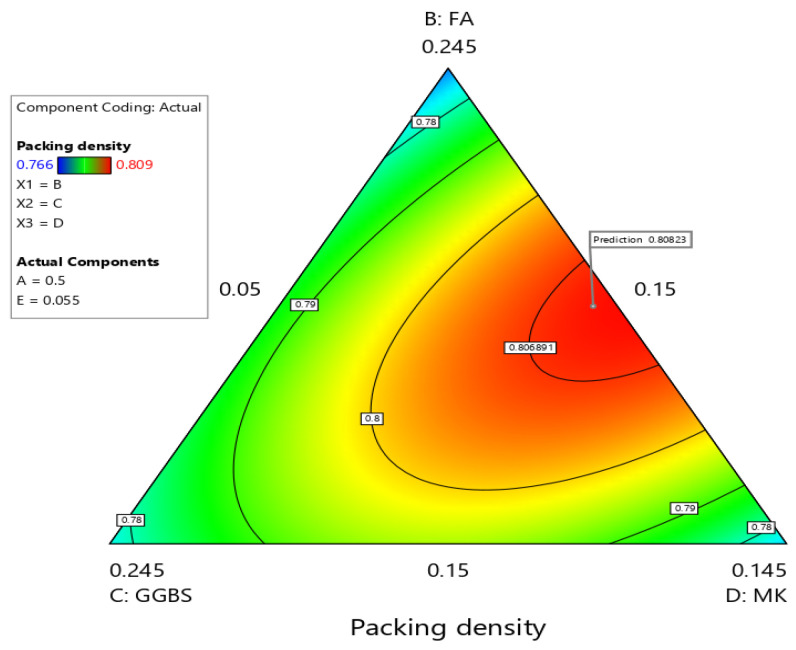
Contour plot showing packing density of MCB.

**Figure 9:**
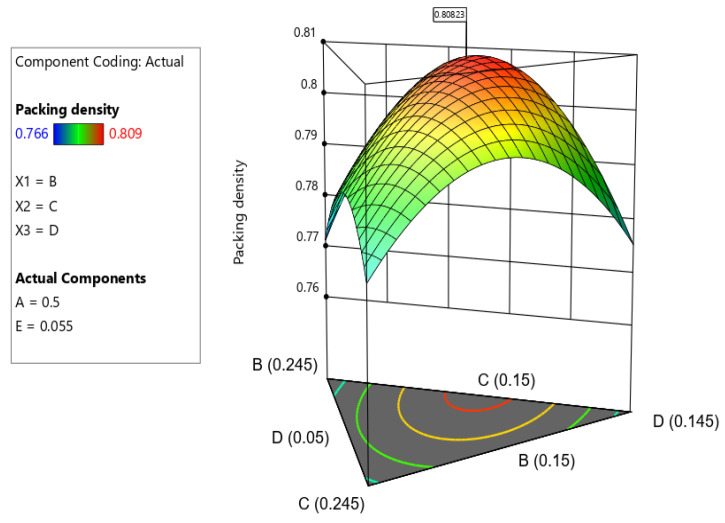
3D surface plot showing packing density of MCB.

In the DOD method, the effect of variations in different components on the response value is represented by the sparsity of the contour lines and the curvature of the 3D response surface. A sharper curvature in the response surface suggests a stronger interaction between components, while a higher density of contour lines indicates a greater significance of the components. Figure 8 displays the contour plot, which reveals interactions between the components. As the SF content increases, the contour lines become more compact and denser, indicating a substantial effect on packing density. This indicates that finer particles in SF disrupt the packing arrangement, leading to a reduction in packing density. A curved surface shown in Figure 9 suggests that the interaction between the components significantly influences the outcome. The curvature might imply that increasing one component has a different effect depending on the level of the other component. If the surface is highly curved, it shows that the components have an interactive effect on the response. For example, increasing SF content might reduce packing density significantly only when fly ash is above a certain level. This highlights the importance of tuning the mix design to find the right balance among components. When these SCMs are proportioned appropriately, a multi-scale packing structure is formed, where larger particles create the skeletal framework, micron-sized particles fill interparticle voids, and nano-sized particles fill submicron gaps. This hierarchical filling effect leads to a nonlinear enhancement in packing density, which is reflected as the high curvature observed in the response curved surface.

The experimental findings are compared with the predicted value, and the absolute relative deviation (ARD) is used to evaluate the precision of the developed model. ARD (%) is computed using Equation (10)

(10) $$\mathrm{ARD}\;(\%)=\frac{Experimental-Model}{Experimental}\times 100$$

In this study, the developed model was further validated by the fact that there was an absolute relative difference of 0.874 between the predicted value and the actual result (see Table 6). In the context of optimizing SCM content while reducing OPC, the reasonable response (packing density) will act as the target outcome to ensure that the mix remains practical and effective. Thus, maintaining a high packing density is essential to ensure good particle packing and reduce voids in the mix.

### 5.2. Optimization of Fine Aggregate in Cement Mortar by Using Particle Packing Methods

#### 5.2.1. Particle Packing Methods (PPMs) for Proportioning of Fine Aggregate

The proportioning of fine aggregates is performed by adopting MTM, JDD, and IS 650:2008, and the resulting proportions are then compared as specified by IS 383 [44].

**Figure 10:**
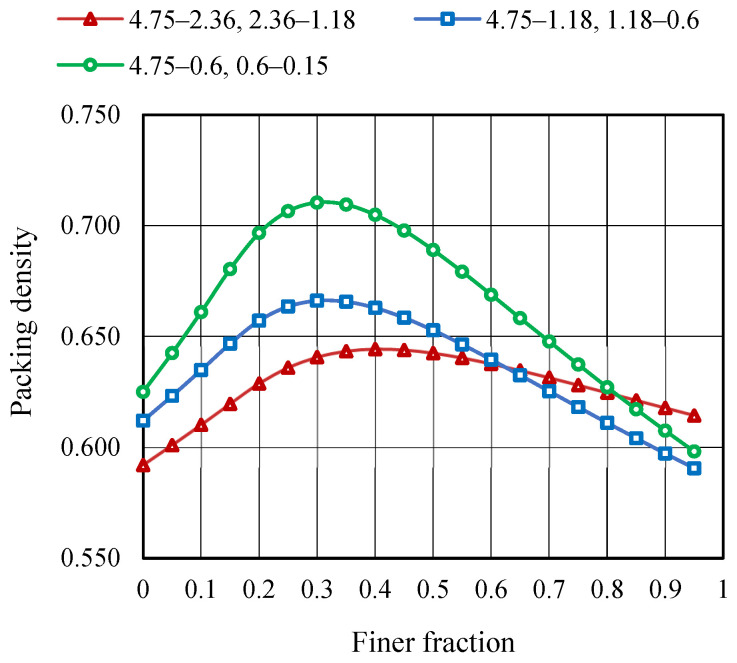
Packing density curve—MTM method.

**Figure 11:**
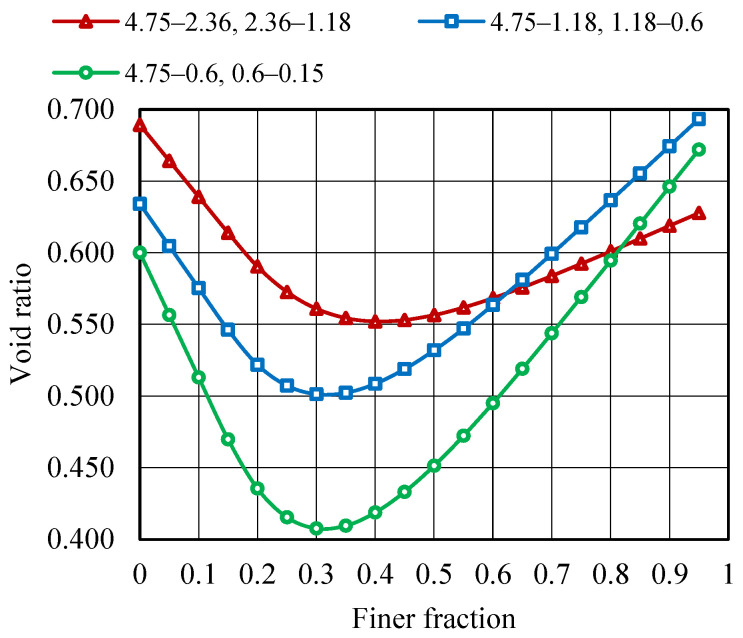
Void ratio curve—MTM method.

Modified Toufar Model (MTM) Method:

MTM approach is used to obtain the highest possible packing density by blending aggregate fractions of various sizes. The initial properties of the fine aggregate fractions, such as specific gravity (G), bulk density (γ), and packing degree (φ), are determined and are presented in Table 7. Aggregate size is varied within the ranges of 4.75–0.15 mm to determine the maximum packing density ($\varphi_{max})$ and corresponding minimum void ratio ($e_{min})$. As shown in Figure 10 and Figure 11, the $\varphi_{max}$ for various aggregate combinations is 0.710, and the corresponding $e_{min}$ is 0.407. It can be observed that when compared with binary mixing, blending polydispersed aggregates leads to a higher packing density and the lowest void ratio.

**Figure 12:**
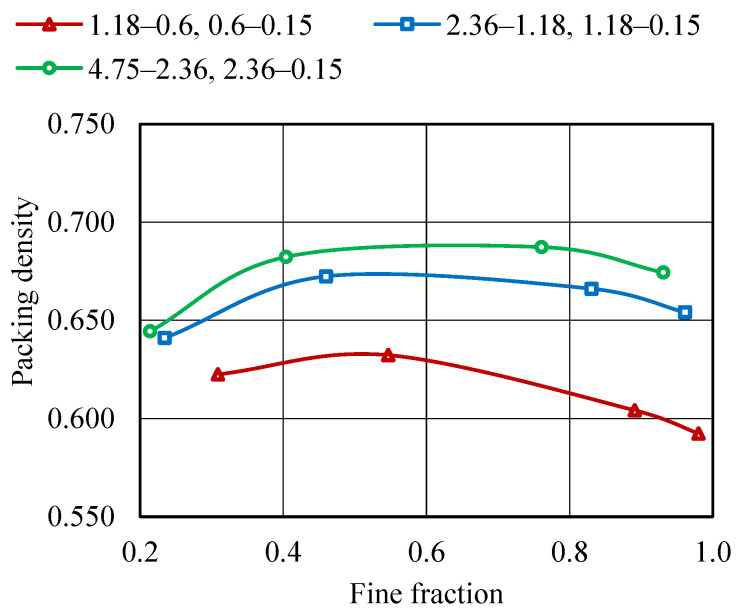
Packing density curve—JDD method.

**Figure 13:**
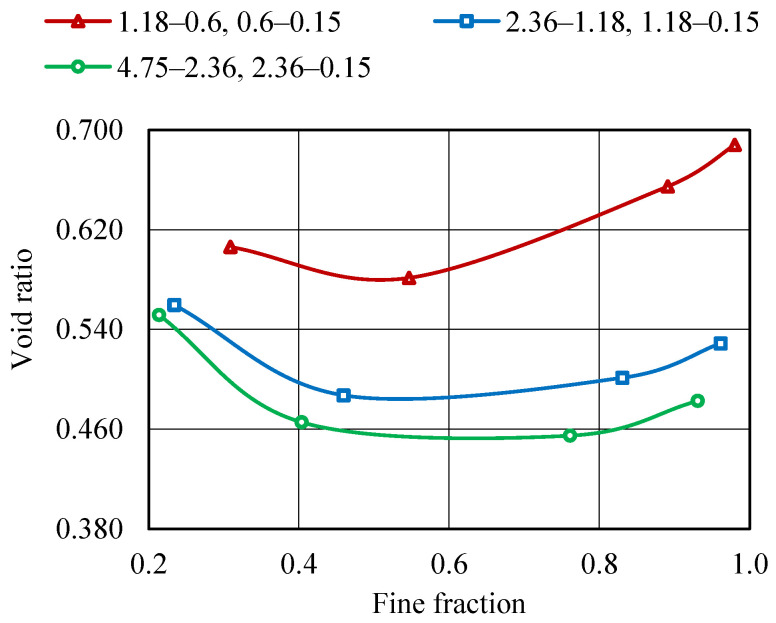
Void ratio curve—JDD method.

J. D. Dewar (JDD) Method:

To achieve a minimum void ratio, the fine aggregate fractions of varying sizes are blended together. The initial properties, such as the G, γ, and e of individual aggregate fractions, are determined and presented in Table 7. $e_{min}$ and $\varphi_{max}$ are determined by altering the sizes of aggregates within a range of 4.75–0.15 mm in various combinations. $\varphi_{max}$ is found to be 0.687 and the corresponding $e_{min}$ for various combinations of aggregates is found to be 0.455, as shown in Figure 12 and Figure 13. Blending two sizes of aggregates, 1.18–0.15 mm and 0.6–0.15 mm, results in a larger void ratio for finer aggregate fractions as the finer fraction is increased. This phenomenon can occur when there is a significant presence of fine aggregate particles in larger proportions, resulting in an aggregate system with a total porosity above 0.5. Nevertheless, the combination of aggregates of various sizes results in enhanced packing density and a decreased void ratio.

IS 650:2008 Method:

As per IS 650:2008 [41], the mortar cubes should be prepared using equal fractions from three distinct fine aggregate size ranges. Table 7 illustrates the packing densities and void ratios of aggregate sets when they are combined in equal quantities. It is worth noting that when equal amounts of aggregate fractions are taken into account, the packing density achieved is not $\varphi_{max}$, and $e_{min}$ achieved is not the least. It is clear from this study that an equal distribution of fine aggregate proportions does not lead to improved packing. The void ratio and packing density attained for equal quantities of aggregates in accordance with IS 650:2008 [41] are 0.534 and 0.652, respectively. PPM approaches produce aggregate fractions that are more uniformly distributed, resulting in densely packed aggregate matrices with a $e_{min}$ and $\varphi_{max}$ when compared with IS 650:2008 [41].

**Figure 14:**
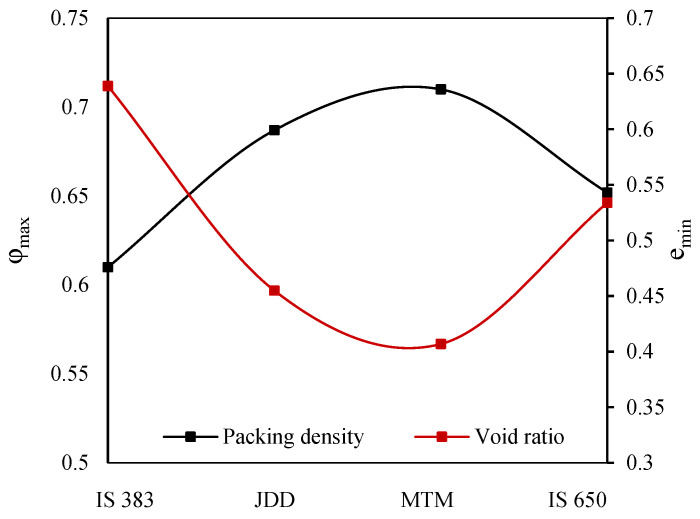
Packing density vs. void ratio of fine aggregate using PPMs.

The proportioning of fine aggregates is performed by adopting MTM, JDD, and IS 650:2008 methodologies, and then the resulting proportions are compared as specified by IS 383 [42]. The $e_{min}$ and $\varphi_{max}$ that are determined using the MTM, JDD, IS 650, and IS 383 methods are shown according to their respective values in Figure 14. Comparatively, the MTM approach showed the highest packing density (0.71) and the lowest void ratio (0.407) when compared with other methods that are considered. Furthermore, the IS 383 approach yielded the lowest packing density (0.61) with the highest void ratio (0.639). It is clear that the PSD suggested by IS 650 and IS 383 does not consider packing density as a factor.

**Figure 15:**
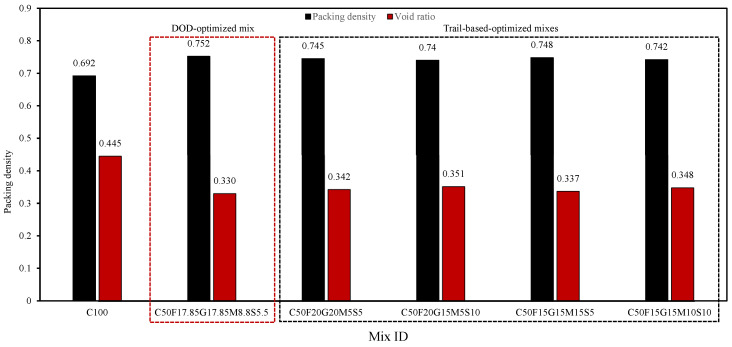
Packing density and void ratio—C100 and CFGMS mortar using WPM.

Packing density and void ratio of mortar using WPD:

The MTM method specifies a particular proportion of sand to be mixed with cement to create mortar. Further, the WPD method is used to determine the $\varphi_{max}$ and $e_{min}$ of mortars, and the resulting values are shown in Figure 15. It involves filling a container with the material in a wet state and then measuring its volume and mass to determine the packing density and void ratio. Figure 15 displays the packing density and void ratio of C100 and CFGMS mortar mixes using WPD, and these parameters are essential for optimizing the mixture design and understanding the behavior of cementitious systems. It is observed that the control mix (C100) shows moderate packing density due to the absence of SCMs, which can improve particle arrangement when compared with other mixes. The best mix in terms of packing density is C50F17.85G17.85M8.8S5.5 due to the optimized proportion of SCMs and sand, which complements particle sizes and shape.

#### 5.2.2. Strength Development: Compressive and Flexural Strength of Cement Mortar

Strength tests on SCMs were conducted to maintain a particular range of workability (or) flow, which leads to different water contents for different pozzolans. In this study, the mortar mixes were maintained with a water-to-binder ratio of 0.45 for consistency. The workability of the mortar mixes evaluated using the flow test (using a mini slump cone with a 70mm top diameter, 100mm bottom diameter, 60 mm height, and 25 blows), ASTM C1437 [62], was reported to be in the range of 150–170 mm without the addition of SP, indicating moderate workability. With the addition of SP 1% (by wt. of binder), the flow improved significantly to 190–225 mm, demonstrating its effectiveness in enhancing the workability of the mixes.

**Figure 16:**
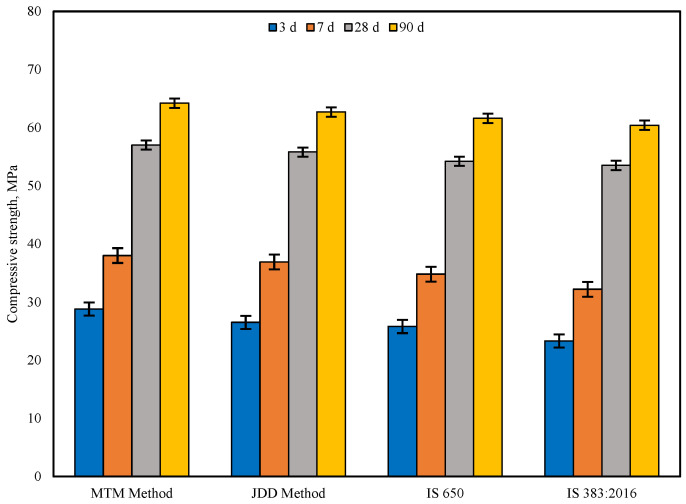
Compressive strength of C100 mortar.

The compressive strength of cement mortar specimens at 3, 7, 28, and 90 days was studied initially for conventional mortar (C100) incorporating aggregate proportions specified in IS 383:2016, IS 650:2008, MTM, and JDD. It is evident from Figure 16 that the MTM approach yields maximum packing density and the highest compressive strength at all ages of curing compared with other methods due to its ability to achieve dense packing of aggregates within the mixture. The compressive strengths of mortar based on JDD, IS 650:2008, and IS 383:2016 samples at 7 days are 2.98%, 9.19%, and 18.01% lower when compared with the MTM method, respectively. JDD, IS 650:2008, and IS 383:2016 exhibit 2.15%, 5.16%, and 6.54% decreases in comparison with the MTM method at 28 days. Similarly, the JDD, IS 650:2008, and IS 383:2016 exhibit a 2.39%, 4.22%, and 6.29% decrease in comparison with the MTM method at 90 days. The MTM method, which achieves a denser packing of aggregates in cement mortar (C100), was selected to further achieve the compressive strength of multi-component mortar mixes.

**Figure 17:**
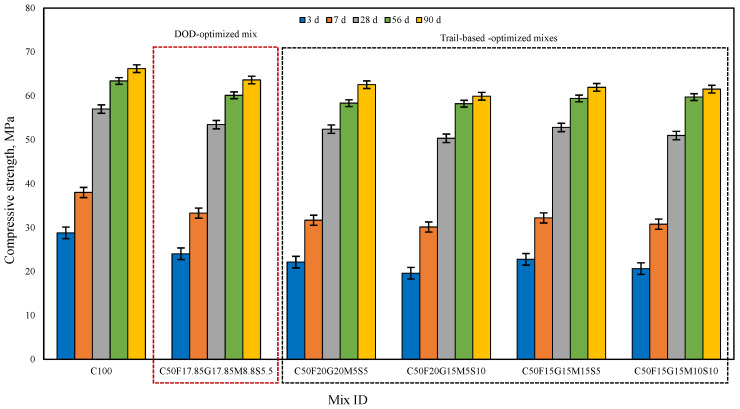
Compressive strength of C100 and CFGMS mortars.

**Figure 18:**
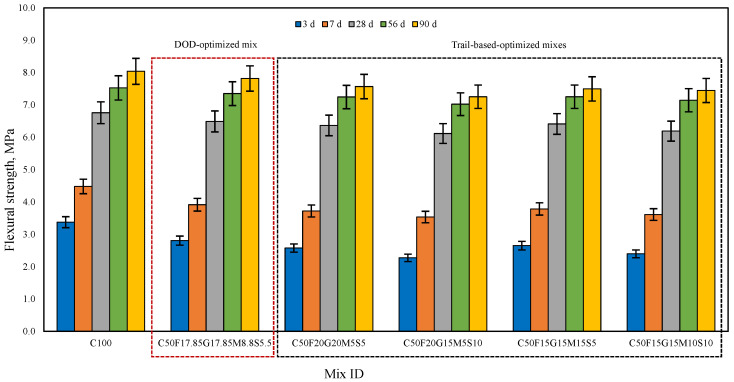
Flexural strength of C100 and CFGMS mortars.

The compressive and flexural strength of cement mortar specimens at 3, 7, 28, 56, and 90 days were studied for C100 and CFGMS mix combinations, incorporating aggregate proportions obtained as per the MTM method. The data presented in Figure 17 and Figure 18 compare the compressive and flexural strength development of C100 and CFGMS over time. However, the specific performance varies based on the precise SCMs adopted and the proportion of replacement employed.

C100 achieves higher early strength, reaching 57 MPa (compression) and 7.65 MPa (flexure) at 28 d, whereas C50F17.85G17.85M8.8S5.5 (DOD-optimized mix) shows slower early strength, reaching 53.21 MPa (compression) and 7.27 MPa (flexure) at 28 d. The high early strength of C100 is primarily due to rapid hydration of OPC, which generates the majority of strength within the first 28 d, while the early strength development of CFGMS is slower than C100, which is designed for long-term performance. The trial mix combinations C50F20G20M5S5, C50F20G15M5S10, C50F15G15M15S5, and C50F15G15M10S10 show similar compression and flexural strength levels, indicating that the varied proportions of the CFGMS provide comparable performances. CFGMS mixes exhibit an increase in compressive and flexural strength at the later ages of 28 d and 90 d that are either nearly identical or slightly higher than those of the control mix (C100). The progressive strength evolution, particularly for the mixes containing SCMs, is attributed to pozzolanic reaction, which typically enhances with the advancement of curing age [63]. Over time, the pozzolanic properties of SCMs become more prominent. This means that they react with calcium hydroxide (CH) produced during cement hydration to form additional binding materials, such as calcium silicate hydrate (C–S–H) gel. However, due to improved particle packing, the SCMs contribute to long-term strength by producing secondary hydration products like C–S–H, which further densifies the microstructure and improves strength at later stages (28 d and beyond). A combination of OPC and SCMs may result in synergistic effects, where the properties of individual components complement each other. These synergistic effects can lead to improved particle packing and densification of the mixes. The compressive strength of C100 will likely continue to increase slightly, reaching around 66.2 MPa at 90 days, as most of the cement hydration is already complete by 28 d. This increase will be marginal because the hydration process of cement slows down significantly after 28 d. The compressive strength of the CFGMS mix will see more substantial gains between 28 d and 90 d. This is due to the slower reactivity of FA and GGBFS, which contribute to strength development over a longer period. By 90 d, the compressive strength is likely to reach 63.62 MPa, approaching or even matching the strength of the C100 mix. The addition of ultra-fine materials like SF and MK improves particle packing and helps in reducing voids. This leads to a denser microstructure, which contributes to enhanced long-term strength. The presence of SCMs helps reduce the overall porosity of the mortar, making it more impermeable. This reduction in porosity contributes to both compressive and flexural strength gains over time. DOD-optimized mix (C50F17.85G17.85M8.8S5.5) likely offers the best overall balance of compressive and flexural strength, with a well-optimized SCM proportion for both early and long-term performance. Optimized proportions based on trial mixes show specific strengths. Mixes with higher SF (C50F20G15M5S10 and C50F15G15M10S10) show better flexural strength, while those with higher FA or GGBFS (C50F20G20M5S5) surpass in long-term compressive strength. High MK mixes (C50F15G15M15S5) provide robust early-age strength. The relative compressive and flexure strength values at 90 days for MCB-based mortar mixes are as follows: C100 > C50F17.85G17.85M8.8S5.5 > C50F20G20M5S5 > C50F15G15M15S5 > C50F15G15M10S10 > C50F20G15M5S10.

#### 5.2.3. Strength Activity Index (SAI)

**Figure 19:**
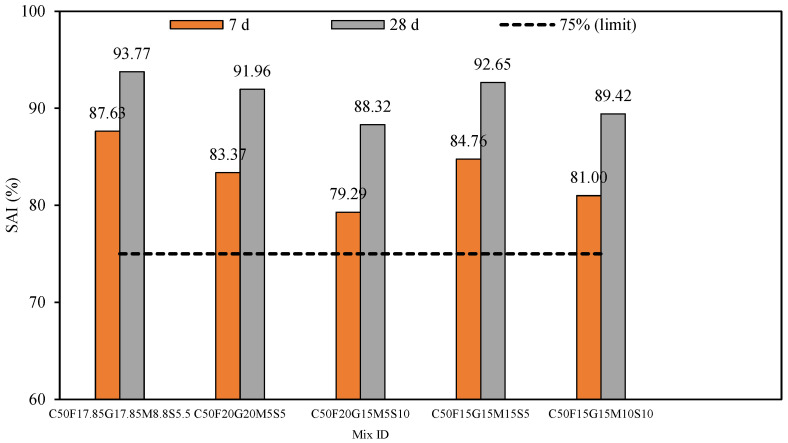
SAI of CFGMS mortars.

In Figure 19, the reference line represents the threshold limit of SAI (≥75%), and if the SAI value exceeds this limit at the age of 28 days, the mix is considered to be pozzolanic. The multi-component mortar systems that correspond to conventional mortar at 7 and 28 days are depicted in terms of their SAI.

Figure 19 clearly shows that the MCB-based mortar mix C50F17.85G17.85M8.8S5.5 had an SAI greater than the recommended limiting value of 75% (as outlined in ASTM C618 even after just 7 d). However, the SAI value was observed to be above 75% after 28 d. This is mainly due to the high reactivity and filler effect of the extremely fine/high surface area of SCMs. Based on experimental results, MK and SF exhibit a twofold mechanism due to their large SSA, i.e., (a) enhanced pozzolanic reactivity and (b) pore filling resulting in denser cementitious systems. Furthermore, the presence of a high proportion of reactive silica content in MK and SF accelerates the pozzolanic reactivity to a great extent. Consequently, this results in improved microstructure owing to the formation of C–S–H. The relative SAI values for CFGMS mortar mixes are as follows: C50F17.85G17.85M8.8S5.5 > C50F15G15M15S5 > C50F20G20M5S5 > C50F15G15M10S10 > C50F20G15M5S10.

#### 5.2.4. Capillary Water Absorption

**Figure 20:**
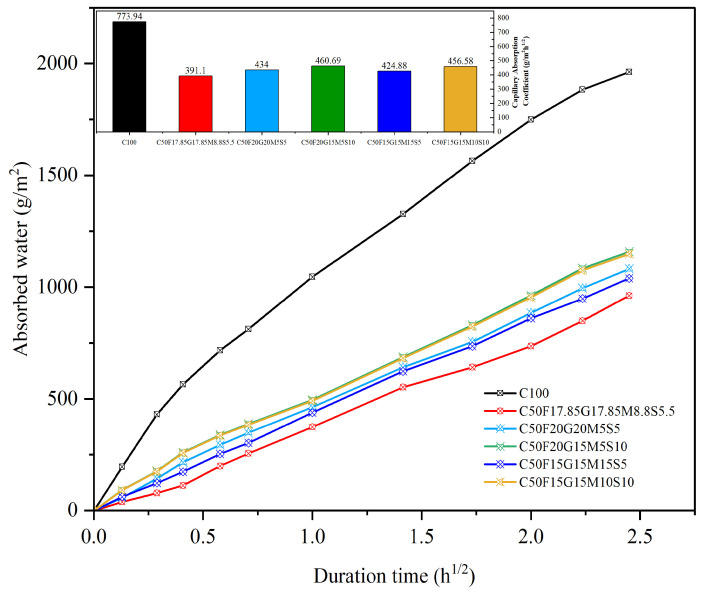
Capillary water absorption of C100 and CFGMS mortars.

The water absorption process is especially important for SCMs, as it contributes to a more durable and impermeable mortar, and the pozzolanic reaction typically becomes noticeable after 56 d. Figure 20 shows the water absorption values of the MCB-based mortar mix CFGMS after 56 days of curing. The amount of absorbed water (capillary water absorption coefficient, g/m^2^h^1/2^) for C50F17.85G17.85M8.8S5.5 (391.1) is less than that of C100 (773.94). This could be because of the pozzolanic and microfiller effect. SCMs may react with calcium hydroxide in cement paste, resulting in additional hydration products that decrease permeability and increase durability. However, the combined effect of the packing density of cementitious materials and fine aggregate minimizes pathways for water to penetrate the mortar, therefore minimizing water absorption.

#### 5.2.5. Drying Shrinkage

**Figure 21:**
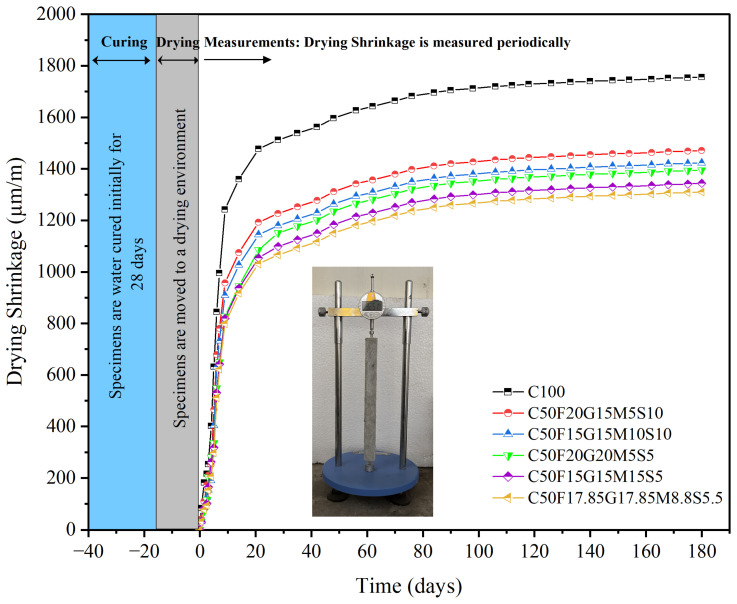
Drying shrinkage of C100 and CFGMS mortars.

Figure 21 depicts the variation in drying shrinkage (µε) at various exposure periods for all the mixes. The drying shrinkage of all the mixtures demonstrates a substantial rate of increase during the initial 14-day exposure period. This is a result of hydration activity, which is induced by the intake of water stored in capillary pores, as well as the further impact of chemical and autogenous shrinkage. Drying shrinkage is the result of excess water evaporating from the capillary pores of hardened cementitious mortar. It is observed that the drying shrinkage of CFGMS mixes shows less shrinkage compared with C100. The C50F17.85G17.85M8.8S5.5 exhibits a drying shrinkage of 1310 µε during the 180-day exposure period, which is less than the control mix (C100) exhibiting 1756 µε. In comparison with C100, the 14-day shrinkage of C50F17.85G17.85M8.8S5.5 decreases substantially from 1360 µε to 917 µε (32.57% reduction), while 180-day shrinkage decreases from 1756 µε to 1310 µε (25.4% reduction). For 14-day shrinkage, the reduction is mainly due to lower cement content, resulting in retardation of hydration, while at 180 days, the reduction is mainly because of reduced capillary pore extent due to better particle packing. By replacing a portion of OPC with SCMs, the total cement content is reduced, which can lead to low levels of drying shrinkage since there’s less hydration from the cement itself. SCMs can improve the binding characteristics of the mix, leading to denser microstructures and a reduction in porosity, which helps mitigate shrinkage. SCMs can contribute to a more efficient water usage in the mix, which may lead to lower drying shrinkage as there is less free water to evaporate. The pozzolanic reactions of SCMs can produce additional cementitious materials over time, contributing to the strength and stability of the matrix, thereby reducing shrinkage further.

### 5.3. SEM Analysis

**Figure 22:**
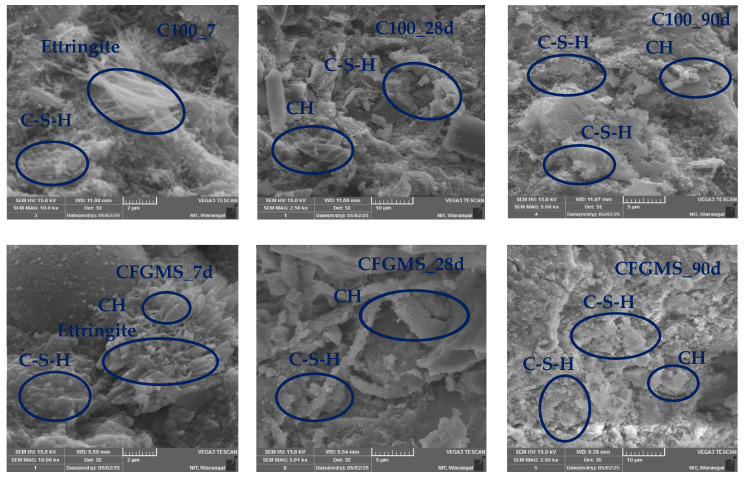
SEM images of C100 and CFGMS.

Figure 22 shows the SEM images of C100 and C50F17.85G17.85M8.8S5.5 mortar mixes corresponding to the MTM method at 7, 28, and 90 days. It is observed that the C100 mortar has a porous microstructure at 7 days, characterized by prominent needle-like ettringite and plate-like calcium hydroxide (CH) crystals. In contrast, the C50F17.85G17.85M8.8S5.5 mortar, incorporating OPC, FA, GGBS, MK, and SF, exhibited early signs of microstructural refinement. The presence of SF and MK initiated early pozzolanic reactions, resulting in a denser matrix and reduced CH formation compared with C100. At 28 days, the C100 matrix showed moderate densification, although CH crystals remained widespread and the interfacial transition zone (ITZ) was still a relatively weak region. Meanwhile, the CFGMS mortar exhibited substantial microstructural development with a compact and continuous matrix composed primarily of amorphous C–S–H gel. The combined pozzolanic activity of these SCMs led to a significant reduction in CH content and refinement of ITZ. At 90 days, C100 continued to show visible CH deposits and retained porosity, while the C50F17.85G17.85M8.8S5.5 mortar achieved a highly dense and homogenous microstructure. SEM images revealed nearly complete CH consumption, a fully developed C–S–H matrix, and an indistinguishable ITZ, indicating strong integration between paste and aggregates. The microstructure exhibits increased density at subsequent ages relative to the initial hydration age. This also signifies the maximum packing density of the binder system in CFGMS, together with a substantial packing density of aggregates attained by the MTM method, both of which facilitate the homogenous dispersion and consistent distribution of hydration products. The C–S–H gel volume increased with hydration age in both C100 and C50F17.85G17.85M8.8S5.5. This will improve the compressive strength, as evidenced by the micrographs. Improved packing density reduces the initial interparticle voids, resulting in a lower capillary porosity after hydration. The reduced interparticle spacing enhances ion diffusion efficiency and provides abundant nucleation sites for hydration products, thereby promoting accelerated and more uniform C–S–H formation. Consequently, hydration products more effectively fill residual pore spaces and densify the matrix. SEM observations confirm a denser and more homogeneous microstructure, with fewer capillary voids. This refined pore structure leads to enhanced mechanical strength. Simultaneously, the reduced pore connectivity limits fluid ingress and ion transport, thereby decreasing permeability and significantly improving durability performance.

## 6. Environmental Impact Assessment

The use of SCMs in cement production often reduces its environmental impact, and this effect becomes more pronounced with increasing SCM levels. The environmental influence of SCMS is primarily evaluated in terms of energy consumption and carbon footprint. In the present study, an environmental impact assessment of MCB-based mortar mixes was conducted to determine the energy consumption and CO_2_ emissions corresponding to varying SCM replacement levels. The inventory data were derived from information provided by a local cement plant and subsequently adjusted to include diesel use associated with transporting raw materials to the laboratory (National Institute of Technology, Warangal, India). The binder materials, including cement and SCMs, were transported from sources located 50 kms away, while the fine aggregate was obtained from a site 5 km from the production facility. The energy consumption and CO_2_ emission factors of each material used in these mixes are taken from the available literature, as shown in Table 8.

**Figure 23:**
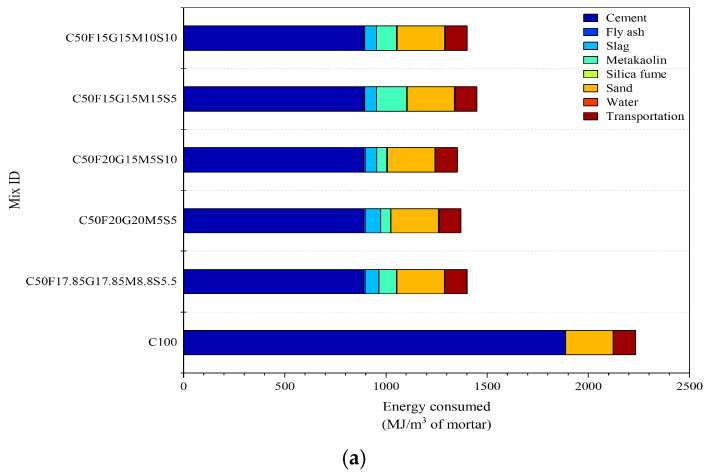

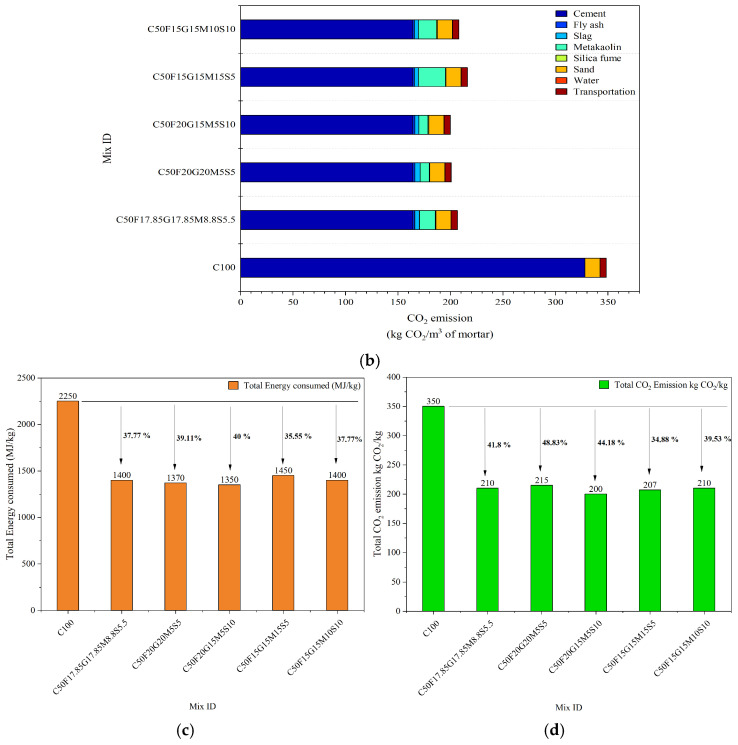
Environmental impact of C100 and CFGMS mortars. (**a**) Energy consumed per m^3^ of concrete; (**b**) CO_2_ emission per m^3^ of concrete; (**c**) Percentage reduction of total energy consumed; (**d**) Percentage reduction of total CO_2_ emission.

Figure 23a,b illustrate the comparative energy consumption and CO_2_ emissions associated with different mortar mixes of MCB. The reference mix (C100), consisting entirely of Portland cement, exhibits the highest embodied energy (2150 MJ/m^3^) and CO_2_ emissions (360 kg CO_2_/m^3^). This is primarily attributed to the high energy demand and carbon intensity of clinker production. In contrast, the CFGMS-based blends demonstrate substantial reductions in both energy consumption and carbon emissions, confirming the environmental benefits of cement replacement with SCMs. Among the blended systems, mixes containing higher proportions of FA and GGBFS, such as C50F20G20M5S5 and C50F20G15M5S10, show the lowest total energy and CO_2_ footprints, ranging between 1350–1370 MJ/m^3^ and 200–215 kg CO_2_/m^3^, respectively. The partial replacement of cement with SCMs markedly reduced both indicators across all blended mixes. Compared with C100, the CFGMS-based systems achieved energy reductions of 35–40% and CO_2_ emission reductions of 34–48%, as shown in Figure 23c. These reductions arise from the lower embodied energy and carbon content of industrial by-products compared with Portland cement. Mixes with relatively higher MK and SF contents (C50F15G15M15S5, C50F15G15M10S10) exhibit slightly elevated values due to the greater processing energy required for their production. In all cases, cement remains the dominant contributor to both energy and CO_2_ impacts, while the contributions of aggregates, water, and transportation are comparatively minor. Overall, the results highlight that strategic incorporation of SCMs, particularly FA and GGBFS, can significantly improve the sustainability of high-performance mortar systems by reducing both embodied energy and greenhouse gas emissions without compromising performance.

## 7. Conclusions

The DOD method provides an effective theoretical framework for designing MCBs with high packing density. Studies were conducted to characterize the properties of cement mortar by proportioning fine aggregate using PPMs (MTM, JDD, IS 650:2008). The conclusions obtained are as follows:

1. Experimental verification confirms that the MCB mix C50F17.85G17.85M8.8S5.5 achieves a high packing density, validating the reliability of the design approach.
2. The particle packing methods (MTM and JDD) enhance packing density and reduce the void ratio. Proportioning of fine aggregate using the MTM method achieves maximum packing density (0.71) and the minimum void ratio (0.407) when compared with the IS 383:2016 and IS 650:2008 methods.
3. MCB-based mortars are able to attain maximum compressive and flexural strengths after 90 days. The packing density and pozzolanic reaction are the primary causes of enhancement in mortar mixtures.
4. Strength Activity Index (SAI) values for all MCB-based mortars fulfill the requirement of ASTM C 618 by achieving a threshold limit of SAI (≥75%) at 28 days.
5. Reduction in capillary water absorption of the optimized MCB-based mortars is observed when compared with C100. The combined effect of the packing density of cementitious materials and fine aggregate minimizes the pathways for water to penetrate the mortar, therefore minimizing overall water absorption.
6. The addition of SCMs reduces drying shrinkage of MCB-based mortars. For 14-day shrinkage, the reduction is mainly due to lower cement content resulting in retardation of hydration, while at 180 days, the reduction is mainly because of reduced capillary pore extent due to better particle packing and pozzolanic reaction.
7. The CFGMS-based mortar formulations offer a viable pathway towards low-carbon construction materials. In comparison with C100, the optimized CFGMS systems reduced embodied energy by approximately 35–40% and CO_2_ emissions by 34–48%.

The proposed solutions integrate material, technological, and environmental aspects, providing a novel approach for the design of high-performance and low-emission cementitious binders that address the current challenges of sustainable development in construction.

## Figures and Tables

**Table 2:** Variables and response.

Variables		Constraint Condition		Response	
Components	Coded	Minimum	Maximum		Coded
OPC	X_1_(A)	0.5	0.5	Packing density	y
FA	X_2_(B)	0.15	0.2		
GGBFS	X_3_(C)	0.15	0.25		
MK	X_4_(D)	0.05	0.15		
SF	X_5_(E)	0.05	0.1		

**Table 3:** Experimental runs and responses.

	X_1_(A)	X_2_(B)	X_3_(C)	X_4_(D)	X_5_(E)	Response (y)
1	0.5	0.19	0.15	0.09	0.07	0.806
2	0.5	0.2	0.15	0.1	0.05	0.809
3	0.5	0.2	0.2	0.05	0.05	0.788
4	0.5	0.15	0.2	0.1	0.05	0.792
5	0.5	0.2	0.2	0.05	0.05	0.784
6	0.5	0.15	0.15	0.1	0.1	0.778
7	0.5	0.15	0.19	0.09	0.07	0.798
8	0.5	0.15	0.15	0.15	0.05	0.772
9	0.5	0.2	0.15	0.1	0.05	0.808
10	0.5	0.15	0.2	0.05	0.1	0.774
11	0.5	0.23	0.15	0.05	0.07	0.782
12	0.5	0.25	0.15	0.05	0.05	0.766
13	0.5	0.18	0.18	0.07	0.07	0.798
14	0.5	0.15	0.25	0.05	0.05	0.778
15	0.5	0.17	0.17	0.06	0.1	0.786
16	0.5	0.15	0.2	0.1	0.05	0.795
17	0.5	0.15	0.15	0.15	0.05	0.768
18	0.5	0.19	0.18	0.08	0.05	0.799
19	0.5	0.16	0.22	0.06	0.06	0.785
20	0.5	0.15	0.15	0.1	0.1	0.78
21	0.5	0.17	0.16	0.11	0.06	0.807
22	0.5	0.15	0.23	0.05	0.07	0.779
23	0.5	0.19	0.19	0.05	0.07	0.79
24	0.5	0.2	0.15	0.05	0.1	0.771

**Table 4:** Proportions of mortar mixtures.

Mix Notation (Number Indicates % of Material)	Constituents of Mortar (kg/m^3^)							Remarks
	OPC	FA	GGBS	MK	SF	Sand	Water	
C100	400	0	0	0	0	1200	160	Control mix
C50F17.85G17.85M8.8S5.5	200	71.4	71.4	35.2	22	1200	160	Optimized proportion obtained from DOD method
C50F20G20M5S5	200	80	80	20	20	1200	160	Optimized proportions obtained from trial method
C50F20G15M5S10	200	80	60	20	40	1200	160	
C50F15G15M15S5	200	60	60	60	20	1200	160	
C50F15G15M10S10	200	60	60	40	40	1200	160	

**Table 5:** Analysis results.

Response	Adj-R^2^	Pre-R^2^	Lack of Fit	Model *p*-Value
y (packing density)	0.9337	0.829	3.28	<0.0001

**Table 6:** Developed MCB proportion and packing density.

OPC	FA	GGBS	MK	SF	w/c	Actual Packing Density	Predicted Packing Density	ARD (%)
0.5	0.1785	0.1785	0.088	0.055	0.6	0.801	0.808	0.874

**Table 7:** Proportioning of fine aggregates.

			Monodispersed Aggregates			Polydispersed Aggregates	
Particle Size, d (mm)	Bulk Density, γ (kg/m^3^)	Specific Gravity, G	Packing Density φ	Void Ratio, e	Proportion	Packing Density, φ_max_	Void Ratio, e_min_
MTM method							
4.75–2.36	1492	2.52	0.592	0.689	0.294	0.710	0.407
2.36–1.18	1515	2.48	0.611	0.637	0.196		
1.18–0.6	1483	2.54	0.584	0.712	0.21		
0.6–0.15	1473	2.5	0.589	0.698	0.3		
JDD method							
4.75–2.36	1492	2.52	0.592	0.689	0.21	0.687	0.455
2.36–1.18	1515	2.48	0.611	0.637	0.4		
1.18–0.6	1483	2.54	0.584	0.712	0.17		
0.6–0.15	1473	2.5	0.589	0.698	0.22		
IS 650:2008 method							
4.75–2.36	1492	2.52	0.592	0.689	0	0.652	0.534
2.36–1.18	1515	2.48	0.611	0.637	0.333		
1.18–0.6	1483	2.54	0.584	0.712	0.333		
0.6–0.15	1473	2.5	0.589	0.698	0.333		
IS 383:2016 method							
4.75–2.36	1492	2.52	0.592	0.689	0.027	0.610	0.639
2.36–1.18	1515	2.48	0.611	0.637	0.093		
1.18–0.6	1483	2.54	0.584	0.712	0.53		
0.6–0.15	1473	2.5	0.589	0.698	0.35		

**Table 8:** The energy consumed and CO_2_ emission factors of the raw materials and ingredients.

Materials (kg)	Energy Consumed (MJ/kg)	CO_2_ Emission (kgCO_2_/kg)	Source
OPC	4.72	0.82	Data from different sources Ecoinvent [64], Gettu et al. (2019) [65], Turner and Collins (2013) [66], Kumar et al. (2021) [67]
Clinker	4.45	0.85	
FA	0.1	0.027	
GGBFS	0.94	0.062	
MK	2.5	0.43	
SF	0.1	0.024	
Sand	0.195	0.012	
Water	0.0111	0.000658	
Transportation	1.69	0.092 *	

* kgCO_2_ equation/t-km.

## Data Availability

The original contributions presented in this study are included in the article. Further inquiries can be directed to the corresponding authors.

## Abbreviations

The following abbreviations are used in this manuscript:

CCS	Carbon Capture and Storage
SCMs	Supplementary Cementitious Materials
FA	Fly Ash
GGBFS (or) GGBS	Ground Granulated Blast Furnace Slag
SF	Silica Fume
MK	Metakaolin
PCE	Polycarboxylate Ether
SP	Superplasticizer
DOD	D-Optimal Mixture Design
WPM	Wet Packing Method
MTM	Modified Toufar Model
JDD	J D Dewar Model
MCB	Multi-Component Binders
ANOVA	Analysis of Variance
SAI	Strength Activity Index
